# Influence of the Addition of TiO_2_ Nanoparticles on the Self-Cleaning Capacity of Cementitious Composites

**DOI:** 10.3390/ma17133098

**Published:** 2024-06-25

**Authors:** Carmen Teodora Florean, Alexandra Csapai, Horatiu Vermesan, Timea Gabor, Andreea Hegyi, Vlad Stoian, Willi Andrei Uriciuc, Cristian Petcu, Marius Cîmpan

**Affiliations:** 1 Faculty of Materials and Environmental Engineering, Technical University of Cluj-Napoca, 103–105 Muncii Boulevard, 400641 Cluj-Napoca, Romania; carmen.florean@incerc-cluj.ro (C.T.F.); alexandra.csapai@stm.utcluj.ro (A.C.); timea.gabor@imadd.utcluj.ro (T.G.); 2 National Institute for Research and Development in Construction, Urban Planning and Sustainable Spatial Development URBAN-INCERC Cluj-Napoca Branch, 117 Calea Florești, 400524 Cluj-Napoca, Romania; cristian.petcu@yahoo.com; 3 Department of Plant Culture-Microbiology, Faculty of Agriculture, University of Agricultural Sciences and Veterinary Medicine Cluj-Napoca, 3–5 Calea Mănăstur, 400372 Cluj-Napoca, Romania; vlad.stoian@usamvcluj.ro; 4 Faculty of Dental Medicine, “Iuliu Hațieganu” University of Medicine and Pharmacy, 400012 Cluj-Napoca, Romania; willi.uriciuc@umfcluj.ro; 5 Faculty of Civil Engineering, Technical University of Cluj-Napoca, 15, Constantin Daicoviciu Str., 400020 Cluj-Napoca, Romania; campan_marius@yahoo.com

**Keywords:** TiO_2_ nanoparticles, self-cleaning, self-sanitising, cementitious composites, recycled aggregates

## Abstract

This study evaluated the potential of incorporating TiO_2_ nanoparticles (NT) into cementitious composites to provide self-cleaning and self-sanitising properties, as well as the partial replacement of natural aggregates with recycled glass (RGA), ceramic brick (RBA), granulated blast furnace slag (GBA), and textolite waste (RTA) from electronic equipment on these properties. Based on the research results, the addition of NT to cementitious composites led to a significant reduction in contact angle, which means an increase in surface hydrophilicity. At the same time, Rhodamine B stain fading was highlighted, with the degree of whiteness recovery of NT composites exceeding that of the control by up to 11% for natural aggregate compositions, 10.6% for RGA compositions, 19.9% for RBA compositions, 15% for GBA compositions, and 13% for RTA compositions. In a mould-contaminated environment, it was shown that the introduction of NT allowed the material to develop a biocidal surface capacity which is also influenced by the nature of the aggregates used. Furthermore, the study revealed that, under controlled conditions, certain recycled waste aggregates, such as textolite, promoted mould growth, while others, such as brick and slag, inhibited it, highlighting not just the effect of the addition of NT, but also the significant influence of the aggregate type on the microbial resistance of cementitious composites. These improvements in the performance of cementitious composites are particularly advantageous when applied to prefabricated elements intended for the finishing and decorative surfaces of institutional (schools, administrative buildings, religious structures, etc.) or residential buildings.

## 1. Introduction

In accordance with contemporary regulatory frameworks in the global construction industry, it is of the utmost importance that built heritage fulfils not only mechanical strength and stability standards but also ensures elevated levels of hygiene for occupants. This is coupled with the imperative of enhancing construction durability, practicing responsible utilisation of natural resources, and mitigating environmental impact. A potential resolution to these challenges may be found in the advancement of innovative cementitious composites. These composites should facilitate the recycling of waste and industrial by-products while exhibiting “smart” attributes, such as self-cleaning and self-sanitising capabilities, as well as having resistance to microbial colonisation [1].

The scientific literature indicates various materials [2,3,4,5,6] which, when incorporated into the cementitious composite matrix, contribute beneficially by inducing evident modifications at micro- and nanostructural levels [7]. Research into the use of nanoparticles to improve the overall properties of cementitious composites began in the 1980s and has continued for nearly two decades. A variety of nanoparticles have been studied in this scope, encompassing nano titanium dioxide, nano aluminium dioxide, nano iron oxide, nano zinc oxide, and nano-silica, due to their prominence in various fields, including engineering, food science, and medicine. Among these materials, nano-SiO_2_ is the most commonly used, and its role in inducing structural changes in cementitious composites (when combined with FA or other materials), thereby improving their physico-mechanical properties in both the fresh and hardened state, has been extensively documented in the literature [8].

An illustration of improved mechanical properties through the incorporation of nano-SiO_2_ is provided by Amin and Abu el-Hassan [9]. They conducted a comparative study of the compressive strength of high-strength cements containing nanoferrite and nano-SiO_2_. Their results indicate that samples with 1–5 wt% nano-SiO_2_ exhibited significantly higher compressive strength, approximately 10% greater than those containing nano-Ni and nano-Cu-Zn ferrite. Another study by Zhao et al. [10] highlights the enhancement of frost resistance and compressive strength in normal concrete by the addition of nanosilica (0–20 wt%), with an improvement of up to 20%. According to Shaikh and Supit [11], the incorporation of 1% CaCO_3_ nanoparticles in fly ash concrete resulted in a 22% increase in compressive strength compared to cement mortars. Meanwhile, Wu et al. [12] conducted a study comparing the effects of nano-CaCO_3_ and nanosilica on the strength of high-strength concrete. Their research highlights a sustained and significant increase in the strength of samples containing a combination of nanosilica and ultra-high-strength concrete (UHSC) over a period of 7 days. Conversely, UHSC containing nano-CaCO_3_ shows consistent strength between days 3 and 7, followed by a rapid increase thereafter. Another study by Ren et al. [13] explores the use of nano-TiO_2_ and nano-silica as partial replacements for cement (1%, 3%, and 5% by weight) in conventional concrete. The study highlights that the incorporation of 3% nano-silica and nano-TiO_2_ results in a corresponding increase in compressive strength.

However, the imperative to enhance the characteristics of construction materials, especially cementitious composites, extends beyond solely focusing on their physico-mechanical properties. Utilising the specific attributes of TiO_2_ nanoparticles (NT), which, when exposed to natural or artificial UV radiation, transition into a metastable state, catalysing alterations in the interaction dynamics of water droplets on the cementitious surface, imparting it with superhydrophilicity, and prompting redox reactions capable of breaking down organic molecules, presents a potential avenue for imbuing cementitious composite surfaces with these “smart” characteristics [14]. In the case of inorganic substances adhered to the composite surface, the superhydrophilicity mechanism is sufficient to enable self-cleaning capability. This is achieved by the water film collecting and removing the inorganic particles from the composite surface. However, when dealing with organic substances, which include molecules of considerable volume and microorganism cells, the superhydrophilicity mechanism must be supplemented by the capacity for degrading organic molecules and/or cells [14,15,16]. These three mechanisms, all grounded in the behaviour of TiO_2_ nanoparticles under UV radiation, interrelate and reinforce each other. Consequently, the mechanism responsible for augmenting the hydrophilicity of cementitious composite surfaces containing TiO_2_ nanoparticles under UV exposure conditions can be explained by the elevation of hydroxyl groups (OH^−^), a phenomenon discerned through analytical techniques such as X-ray photoemission spectroscopy (XPS), Fourier transform infrared spectroscopy (FTIR), or nuclear magnetic resonance (NMR) [17,18,19,20,21,22].

**Figure 1:**
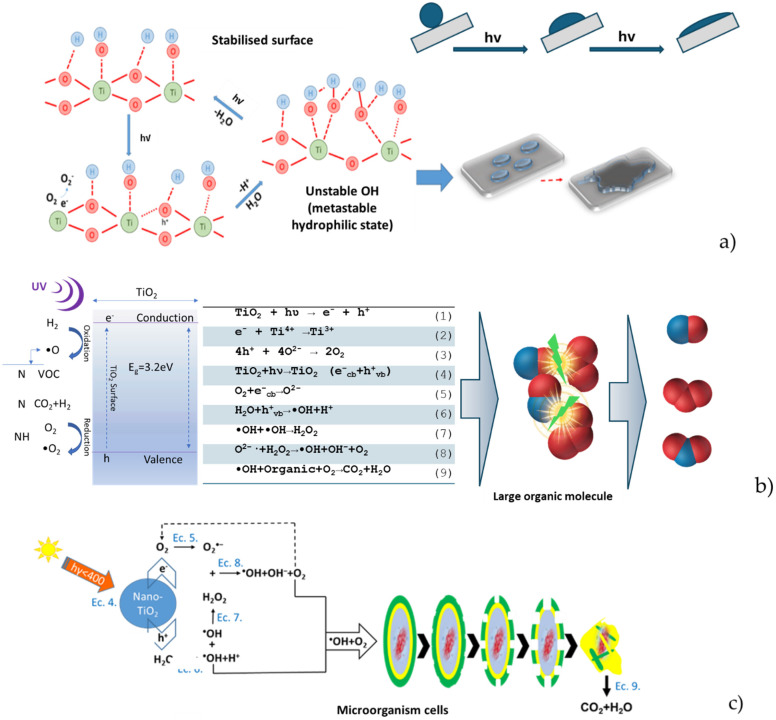
Schematic representation of the self-cleaning mechanism of the surface due to the activation of TiO_2_ nanoparticles under UV irradiation. (**a**) Increase in hydrophilicity (authors’ interpretation according to [25]). (**b**) Destruction of the organic molecule (authors’ interpretation according to [26,27]). (**c**) Destruction of the microbial cell (authors’ interpretation according to [26]).

The transition of the surface into a metastable thermodynamic state under the influence of UV radiation can be attributed to the coexistence of two molecular forms of water: molecular water and dissociated water. In essence, the absorption of energy by titanium dioxide, a semiconductor with a band gap of approximately 3.0 eV, results in the generation of electrons (e^−^) and holes (h⁺) [23]. Electrons tend to reduce the Ti(IV) cation to the Ti(III) ion, and holes oxidise O_2_^−^ anions. This phenomenon liberates oxygen, inducing vacancies on the surface of titanium dioxide. These vacancies facilitate the binding of water molecules, thereby releasing hydroxyl groups (OH^−^) [23]. In cementitious composite surfaces incorporating TiO_2_, the existing body of scientific literature [17,18,19,24] indicates that photogenerated holes (h⁺) induce elongation of bonds within the TiO_2_ lattice, thereby transitioning the surface into a metastable state conducive to the adsorption of molecular water. This process occurs simultaneously with the formation of new hydroxyl groups and the release of a proton, as illustrated in Equations (1)–(3) and depicted in Figure 1b.

The hydroxyl groups generated are less thermodynamically stable, which facilitates the spreading of water droplets to cover a larger area for stabilisation [24,28,29,30], thus achieving the super hydrophilic effect. This effect is characterised by the formation of sheets of water on the activated composite surface, which facilitates the removal of impurities. The generation of electron–hole pairs (e^−^) and vacancies (h⁺) leads to the formation of anionic radicals (O_2_^−^) and (OH) through the reactions with O_2_ and H_2_O. These oxidative species (h^+^, O_2_^−^, and OH) are all highly reactive and contribute to the degradation of organic molecules. Initially, this occurs in compounds with smaller molecules, which are easier to remove from the surface by washing. However, eventually, the complete degradation of the organic residue occurs, resulting in the formation of H_2_O and CO_2_. Additionally, these species contribute to the destruction of microorganism cells [23,31] according to a sequence of chemical reactions shown in Equations (4)–(9) (Figure 1b). The processes occurring at the surface of NT-enriched cement, catalysed by nanoparticle photoactivation, occur concurrently, leading to the acquisition of “smart” attributes such as self-cleaning and self-sanitising capabilities. The potential fabrication of such cementitious materials for construction purposes presents extensive applicative prospects. These extend beyond mere aesthetic alterations, as deposits of contaminants on construction surfaces may have a detrimental impact on structural durability and user health. In particular, deposits of mould, algae, and fungi pose a significant threat to structural integrity [14,32,33].

The explanation of the mechanism behind the degradation of cementitious surfaces due to microbial deposits begins with the study of their ability to produce biogenic acids (such as sulphuric acid and nitrifying acid), which contribute to the dissolution of calcium hydroxide (Ca(OH)_2_) and the hydration–hydrolysis compounds of cement [14]. In order to mitigate this phenomenon, it is recommended that buildings be regularly repaired and renovated. Throughout the years, various biocidal treatments have been investigated as the most common methods to counteract the biodeterioration of cement-based materials. However, many of these approaches have been considered unsustainable due to their high levels of toxicity. In contrast, the use of photocatalytic cementitious materials has proven to be more effective in managing biodeterioration processes compared to traditional biocides [14,34].

Given the imperative for responsible resource management, the recycling of various waste materials and their integration as substitute aggregates for natural counterparts offers a viable strategy to mitigate the significant waste generation on a global scale. Scientific studies documented in the literature indicate that construction waste accounts for an estimated 35% of total global solid waste [1,35]. Material consumption and solid waste generation are closely linked to rapid urbanisation and population growth, with housing demand and material consumption expected to increase until at least 2050 [1,2,3,4,5,6,7,8,9,10,11,12,13,14,15,16,17,18,19,20,21,22,23,24,25,26,27,28,29,30,31,32,33,34,35,36]. It is therefore understandable that the reduction, reuse, and recycling of such waste materials is the focus of scientific attention worldwide. This area of research is currently well documented, particularly with regard to the influence of the incorporation of waste and industrial by-products on the physico-mechanical properties of cementitious composites [37,38,39,40,41,42,43], whether used as recycled aggregates or as additives with pozzolanic properties. Nevertheless, studies on the impact of waste recycling in cementitious compositions, particularly with regard to their resistance to microbial activity [44,45] (such as mould, algae, and lichens) and/or in relation to the potential development of novel composites with “smart” properties (including self-cleaning, self-sanitising, and self-healing properties) [46,47] are relatively scarce in the literature. This shortcoming can be attributed, in part, to the apparent need for interdisciplinary collaboration across fields as diverse as building materials science, civil engineering, environmental studies, biology, microbiology, and even medical sciences.

Therefore, the objective of this study was to evaluate the potential for the incorporation of TiO_2_ nanoparticles (NT) into cementitious composites to provide self-cleaning and self-sanitising properties. This investigation involved the partial replacement of natural aggregates (NA) with aggregates derived from locally recycled waste materials, including recycled glass waste (RGA), recycled ceramic brick waste (RBA), granulated blast furnace slag (GBA), and textolite waste from electronic equipment (RTA).

## 2. Materials and Methods

### 2.1. Raw Materials

The following raw materials were selected for the production of the cementitious composites subjected to the experimental study:

- Portland cement CEM I 52.5 R, characterised by a minimum content of 95% Portland clinker (Table 1) and a compressive strength at 28 days of at least 52. 5 N/mm^2^, produced by HOLCIM Romania, Aleșd, Bihor County, Romania.
- Natural aggregates (NA) with grain size classes 0/4 mm and 4/8 mm; recycled glass aggregate (RGA) with grain size classes 0/4 mm and 4/8 mm; recycled ceramic brick aggregate (RBA) with grain size class 0/4 mm; blast furnace slag (GBA) with grain size class 0/2 mm, characterised in terms of chemical composition according to Table 2; recycled textolite waste aggregates (RTA) (characterised in Table 3), with particle size class 0/2 mm.
- Superplasticiser additive MasterEase 5009 (BASF, Ludwigshafen, Germany), a superplasticiser additive based on polymeric compounds with a water-soluble chloride content of maximum 0.1% (wt.) and an alkali content (Na_2_O equivalent) of maximum 2.5% (wt.), according to EN 934-1:2008 [48] and EN 934-2:2009 [49].
- TiO_2_ nanoparticles of the type AEROXIDE^®^ TiO_2_ P25 (Evonik Degussa Industries AG, Hanau, Germany) characterised by an average particle size of 21 nm, with a specific surface area of 35–65 m^2^/g, a purity of 99.5%, containing more than 70% anatase crystalline phase, and containing water. The Portland cement and all recycled waste aggregates were of domestic origin.

**Table 1:** Characterisation of Portland cement.

	SiO_2_	Al_2_O_3_	Fe_2_O_3_	CaO	MgO	SO_3_	Na_2_O	K_2_O	P_2_O_5_	TiO_2_	Mn_2_O_3_
w%	15.02	3.17	3.01	55.12	3.13	3.74	0.23	1.15	0.12	0.28	0.04
Portland clinker (%) EN 197-1 [50]			Minor component (%) EN 197-1 [50]		Sulphate content (as SO_3_%) EN 196-2 [51]			Chloride content (%) EN 196-2 [51]		Insoluble residues (%) EN 197-1 [50]	
95–100			0–5		≤4			≤0.1		≤5

**Table 2:** Characterisation of blast furnace slag (GBA).

	SiO_2_	Al_2_O_3_	Fe_2_O_3_	CaO	MgO	SO_3_	Na_2_O	K_2_O	P_2_O_5_	TiO_2_	Cr_2_O_3_	Mn_2_O_3_	P.C
w%	30.20	10.05	14.70	37.40	4.05	-	0.20	0.38	-	<0.52	<0.05	2.15	-

Note: It should be noted that the values represented by the symbol < represent values below the limit of determination of the method.

**Table 3:** Characterisation of textolite waste (RTA).

	As	Ba	Cd	Cr	Cu	Hg	Mo	Ni	Pb	Sb	Se	Zn
mg/kg	2.85	603.8	<0.03	18.2	1064	<0.003	<3.0	<0.20	<0.30	<0.5	<0.015	12.59

Note: It should be noted that the values represented by the symbol < represent values below the limit of determination of the method.

### 2.2. Manufacturing of Cementitious Composites and Specimens

**Figure 2:**
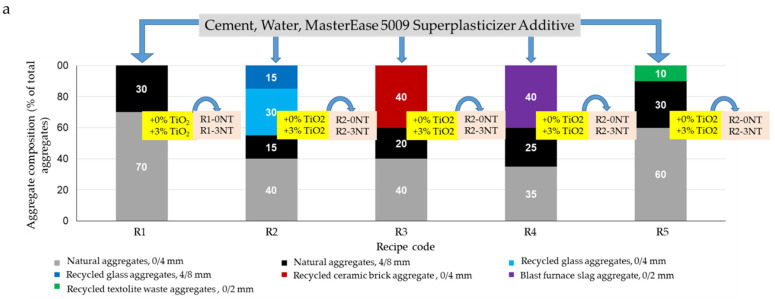

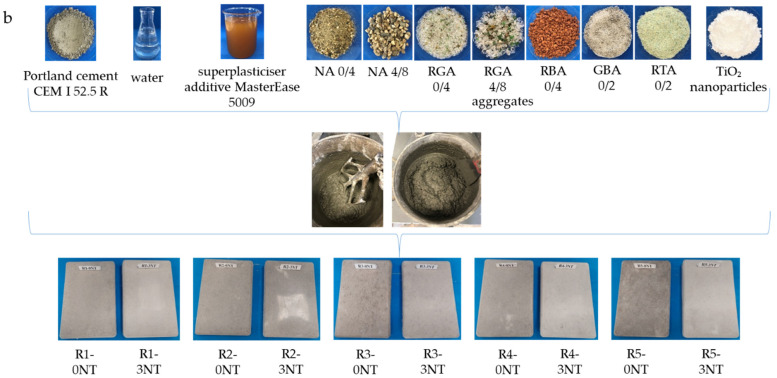
Schematic representations of (**a**) prepared and tested cementitious composite variants; (**b**) the sample preparation process.

For the purpose of this research, 5 sets of 2 compositional variants of cementitious composites were prepared and analysed as follows (Figure 2a,b):

- First set—Cementitious composite comprising cement, natural aggregates, superplasticiser additive, and water, as shown in Table 4, without the addition of TiO_2_ nanoparticles, referred to as the control group (R1-0NT), and with the addition of 3% TiO_2_ nanoparticles (by weight relative to the amount of cement) (R1-3NT).

- Second set—Cementitious composite consisting of cement, natural aggregates partially substituted by recycled glass waste aggregates, superplasticiser additive, and water, as shown in Figure 2, without the addition of TiO_2_ nanoparticles (R2-0NT) and with the addition of 3% TiO_2_ nanoparticles (weight relative to the amount of cement) (R2-3NT).
- Third set—Cementitious composite consisting of cement, natural aggregates partially substituted with recycled ceramic brick waste aggregates, superplasticiser additive, and water, as shown in Figure 2, without the addition of TiO_2_ nanoparticles (R3-0NT) and with the addition of 3% TiO_2_ nanoparticles (weight relative to the amount of cement) (R3-3NT).
- Fourth set—Cementitious composite consisting of cement, natural aggregates partially substituted by waste slag aggregates, superplasticiser additive, and water, as shown in Figure 2, without the addition of TiO_2_ nanoparticles (R4-0NT) and with the addition of 3% TiO_2_ nanoparticles (weight relative to the amount of cement) (R4-3NT).
- Fifth set—Cementitious composite consisting of cement, natural aggregates partially substituted with aggregates from recycled textolite waste recovered from waste electrical and electronic equipment (WEEE), superplasticiser additive, and water, as shown in Figure 2, without the addition of TiO_2_ nanoparticles (R5-0NT) and with the addition of 3% TiO_2_ nanoparticles (wt. based on the amount of cement) (R5-3NT).

The proportions of natural aggregates replaced by recycled waste aggregates and the addition of 3% TiO_2_ nanoparticles (by weight relative to the amount of cement) were determined based on preliminary research [52,53,54]. This research evaluated the influence of these two factors on the physico-mechanical properties and durability of the cementitious composites. The chosen formulation maximises the use of recycled waste without significantly reducing the physico-mechanical performances, while ensuring that an adequate and beneficial amount of TiO_2_ nanoparticles is incorporated. Surface hydrophilicity, self-cleaning ability, and mould resistance were analysed for each of the 10 composition variants.

The amount of cement was kept constant, and the superplasticiser rate was set at 0.5% by weight based on the cement content for all the compositions. The required mixing water was adjusted to ensure workability, maintaining a spread diameter of 175 ± 10 mm on the flow table (EN 1015-2 [55]). Consequently, the additional water required for the partial replacement of natural aggregates with recycled waste aggregates and/or the incorporation of TiO_2_ nanoparticles resulted in a water/cement ratio between 0.60 and 0.65.

For each composition, a set of samples in the form of plates with dimensions of 65 × 85 × 15 mm and a set of samples in the form of tablets with a diameter of 15 mm and a thickness of 0.5 mm were prepared. After casting, the samples were kept in moulds for 24 h in a humid chamber at a constant temperature of (20 ± 1) °C and a relative humidity of at least 90%. They were then removed from the moulds and immersed in water at a temperature of (20 ± 2) °C until they reached an age of 28 days after casting, and they were then kept in the dark. Upon reaching 28 days post-casting, the samples were subjected to 24 h of UV radiation to activate the TiO_2_ nanoparticles. This was achieved using a UV light source emitting in the315–400nm spectrum, corresponding to the UVA band. The UV source was positioned 10 cm above the surface of the samples, resulting in a light flux intensity of 860 lux.

### 2.3. Contact Angle Measurement

In order to analyse the effect of NT addition on the cementitious composites studied, a contact angle measurement test was carried out at the liquid–composite interface. This was carried out using the Drop Shape Analyser DSA100 (KRUSS, Hamburg, Germany) together with the specialised software Advance for data processing and interpretation. The contact angle for an ultrapure water droplet was measured to an accuracy of 0.01°. The 10 µL droplet was deposited perpendicularly onto the sample surface at a rate of 2.66 mL/s in drop mode. This was intended to measure the contact angle of a sessile drop on a solid surface and calculate the surface free energy (SFE). A video image of the drop was analysed, and the contact angle was measured as the angle between the contour of the drop and the line representing the surface (baseline). The SFE of the solid was able to be calculated from the contact angles of different test liquids. The test was carried out at a minimum of three different points on the surface of the cementitious composite sample, with the results reported as the arithmetic mean of the individual measurements. The contact angle analysis, together with the SFE analysis, was carried out at two specific time intervals: at the moment of initial contact (t_0_) and 100 s after contact (t_100_).

In order to determine whether the addition of NT induces a transition to surface hydrophilicity, a comparative analysis was carried out on composite samples prepared from cement, superplasticiser additive, water, and natural aggregates, both with and without the addition of NT. Specifically, samples R1-0NT and R1-3NT (prepared as described in Section 2.2) were analysed. All tests were carried out under controlled laboratory conditions, in the absence of air currents, using samples prepared, conditioned, and exposed to UV radiation as described in Section 2.2.

### 2.4. Analysis of the Surface Self-Cleaning Performance of Cementitious Composites

For each type of cementitious composite, the initial degree of whiteness was assessed using a type ML-WSB-1 leucometer (PCE Instruments UK Ltd., Hamble-le-Rice, UK) under controlled conditions of constant illumination and light incidence on the surface analysed. The degree of whiteness is defined on a scale from 0 to 100 measurement units (MU), with absolute black having a value of 0 MU and absolute white having a value of 100 MU.

**Figure 3:**
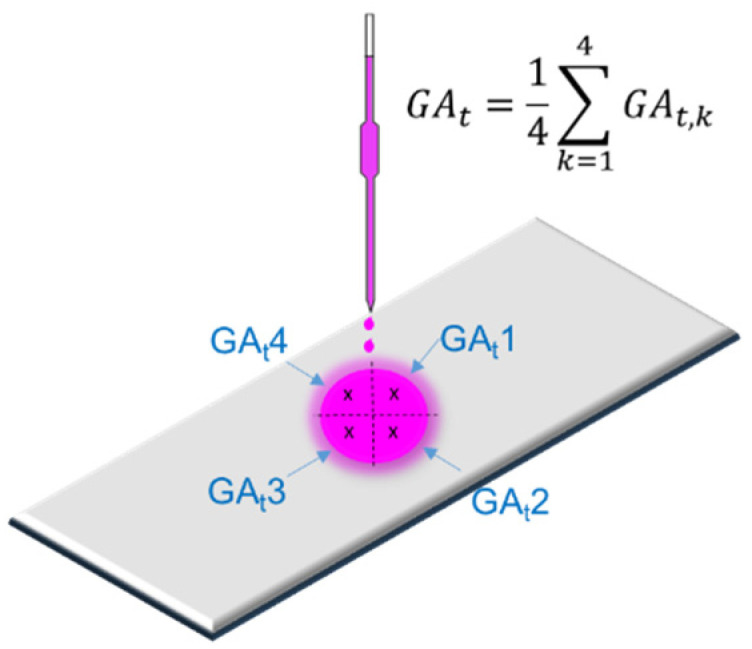
Methodology for self-cleaning assessment.

Subsequently, 0.15 mL of a 1 g/L Rhodamine B solution was applied dropwise to the sample surfaces. The degree of whiteness in the stained area was then measured again and reported as the arithmetic mean of four values, one from each quadrant of the stain, as shown in Figure 3.

**Figure 4:**
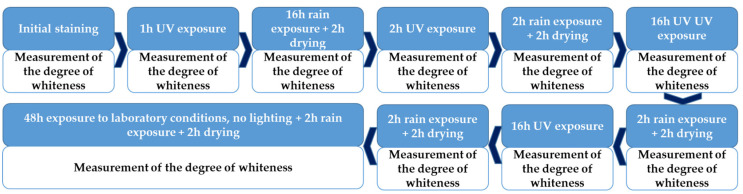
Self-cleaning test cycle.

Following the staining process, the samples were subjected to a test cycle consisting of successive phases of UV exposure, washing under artificial rain, and drying, as shown in Figure 4.

After each step of the cycle, the degree of whiteness in the stained area was measured by averaging four individual measurements at predefined locations. The stained area was also examined microscopically using a LEICA SAPO microscope (Leica Microsystems, GmbH, Wetzlar, Germany).

The exposure to simulated rainfall and subsequent drying was carried out in the absence of any illumination, with the samples inclined at an angle of 30° relative to the vertical axis.

The efficiency of the incorporation of TiO_2_ nanoparticles in improving the self-cleaning properties of the cementitious composite was evaluated both visually and through the following quantifiable parameters:

- The degree of whiteness of the sample—DW—in the stained area, at initial staining and after completion of one or more steps of the testing protocol;
- Recovery of degree of whiteness—represents the proportion of the initial degree of whiteness retained by the sample after one or more of the steps outlined in the testing procedure, expressed through Equation (1):

(1) $$CR=\frac{{GA}_{t}}{{GA}_{0}}*100(\%)$$

where *CR* represents the ability to recover the degree of whiteness, ${GA}_{t}$. The degree of whiteness of the sample within the stained area at stage p of the testing protocol is expressed in units of measurement (UM) on a scale of 0 to 100, where 0 corresponds to absolute black and 100 to absolute white, and ${GA}_{0}$ represents the initial degree of whiteness, of the unstained samples, expressed in units of measurement (MU) on the same 0–100 scale.

### 2.5. Analysis of the Mould Resistance of Cementitious Composite Surfaces

The assessment of mould resistance was carried out on cementitious composites containing natural aggregates (R1) and those containing a partial replacement of natural aggregates with recycled waste aggregates (R2–R5). This evaluation included both compositions with and without the inclusion of NT, with the latter representing 3% of the total mass relative to the cement content.

Two mould strains, namely, *Aspergillus niger* (FAg18003) and *Penicillium chrysogenum* (FAg19002), were selected from samples collected from the interior surfaces of buildings in Cluj, Romania. These strains underwent isolation, purification, and subsequent preservation within the microorganism collection managed by the Microbiology Department of the Faculty of Agriculture, USAMV Cluj-Napoca, Romania. These mould strains were selected for this study because of their prevalence in the built environment, both indoors and outdoors. In addition, their selection was based on the toxicity associated with the aflatoxins emitted and their significant correlation with reported illnesses in the population as a result of exposure [20,56,57,58].

**Figure 5:**
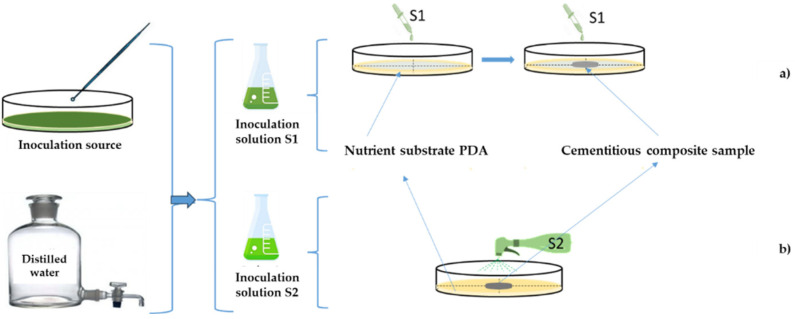
Schematic representation of the working principle for assessing resistance to mould attack: (**a**) application of concentrated contaminating solution by dripping; (**b**) application of diluted contaminant solution by spraying.

The uncontaminated cultures of *Aspergillus niger* (FAg18003) and *Penicillium chrysogenum* (Fag19002) were used as inoculum reservoirs within experimental setups arranged in 9 cm diameter Petri dishes. These vessels were pre-charged with sterile potato dextrose agar (PDA) medium (39 g/L) and subjected to a 4 h sterilisation process under UV irradiation. For each fungal species, *Penicillium chrysogenum* and *Aspergillus niger*, two spore solutions were prepared: The first solution, designated S1, involved the collection of spores from pure fungal cultures, followed by the addition of two aliquots of 10 µL of the biological material to 1 mL of distilled water. A second solution, designated S2, was then prepared by diluting the first solution tenfold by adding two aliquots of 10 µL of the biological material to 10 mL of distilled water. For each mould species, two batches of Petri dishes containing sterilised PDA culture medium were prepared as follows:

- For the S1 inoculation solution, a batch of Petri dishes was prepared by dripping 1.5 mL of the S1 mould spore suspension (Figure 5a), ensuring that the entire PDA surface was covered. A centrally located sample of photoactivated cement composite with nanoparticle additives was then placed in the Petri dish under conditions that prevented cross-contamination. A further 0.5 mL of the spore suspension was then applied to the cement composite sample. The Petri dish was covered with its lid, and the entire system was sealed around the edges with sealing tape to prevent cross-contamination.
- For the S2 inoculant, a batch was prepared by spraying 0.5 mL of the S2 inoculant directly onto the surface of the cement composite sample already placed on the nutrient substrate (Figure 5b). The spraying was performed centrally, perpendicular to the surface and from a constant height of 50 mm, ensuring that the inoculation solutions were distributed both on the surface of the sample and on the uncovered surface of the nutrient substrate. Consequently, in comparison to the concentrated solution (S1), the degree of contamination in this case would theoretically be reduced by a factor of 60 (1/6 of the amount used in the droplet application multiplied by a factor of 10 dilution).

This approach, involving variations in the concentration of the inoculation solution and the method of application, was chosen to highlight the importance of the concentration of the biological contaminant (mould) on the performance of the cement composites. A growth control system was prepared for each batch of Petri dishes and each mould species. These systems did not contain a cement composite sample and were used as controls to verify the viability of the spore solutions used.

The prepared and sealed test systems were placed under laboratory conditions of (23 ± 2) °C, (65 ± 5)% relative humidity, and natural lighting. At predetermined time intervals (24 h, 48 h, 72 h, 30 days, and 120 days—4 months after exposure of the sample in the contaminated system), the samples were examined both visually and microscopically for any signs of growth or development of biological material. The presence and development of the inhibition halo was monitored, and its diameter (D) was recorded as the average of at least four measurements.

## 3. Results and Discussions

### 3.1. Surface Hydrophilicity Analysis of Cementitious Composites

**Figure 6:**
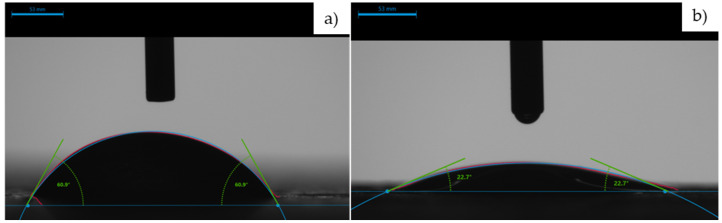
Screenshot image (Speed Camera–DSA100, KRUSS, Germany) at the moment of initial contact between the liquid droplet and the surface of the sample to be analysed: (**a**) R1-0NT; (**b**) R1-3NT.

As illustrated in Figure 6b, at the initial contact moment between the liquid droplet and the analysed sample surface, a flattened droplet formed on the NT-containing cementitious surface, R1-3NT, with a contact angle of 22.7°.

In contrast, on the NT-free cementitious surface, R1-0NT, the droplet exhibited a contact angle of 60.8° (Figure 6a). This reduction in the contact angle by 31.8°, or 62.7% compared to the control sample R1-0NT, provides evidence that the incorporation of NT in the composite matrix, under UV activation, enhances the surface hydrophilicity. Furthermore, a transition from a slightly hydrophilic surface (R1-0NT) to a hydrophilic surface (R1-3NT) was observed.

An analysis of the contact angle evolution over the 100 s testing period, as illustrated in Table 5, revealed a decrease in the angle over time.

**Figure 7:**
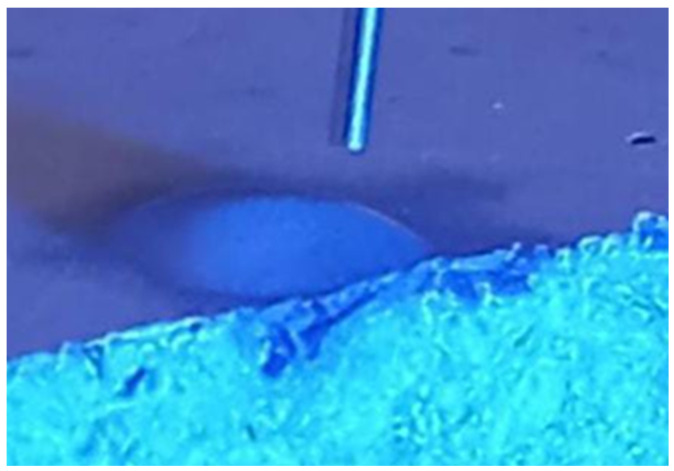
Illustrative image of the ultrapure water droplet on the surface of the cementitious composite and its absorption into the upper layer of the sample.

This trend can be attributed to two phenomena: water evaporation and, more notably, water absorption by the composite, as exemplified in Figure 7.

In comparison, without providing a quantifiable reduction in the contact angle, this phenomenon is reported in the literature by Li et al. [59] for cementitious composites with 3% and 5% NT. Nyong et al. [60] report a reduction in the contact angle of cementitious composites with 1.67% TiO_2_ by 41% after 6 h of UV exposure, 59.6% after 6 h of UV exposure for composites with 3.33% TiO_2_, 64% after 6 h of UV exposure for composites with 5% TiO_2_, and 64.8% after 6 h of UV exposure for composites with 6.67% TiO_2_. These findings confirm the effect of NT on the hydrophilicity of cementitious composite surfaces.

With regard to the quantifiable indicator of surface free energy (SFE), it was observed that at the initial moment (t0), the SFE increased by 42.1% in the presence of NT and UV activation. Following a period of 100 s from the initial contact between the liquid and the sample, the indicator exhibited an increase of 21.4% for the R1-0NT sample and 20.3% for the R1-3NT sample. A higher SFE indicates a more “water-friendly” surface, which is indicative of a greater degree of hydrophilicity. Furthermore, the observation that these indicators underwent change over time throughout the duration of the analysis (100 s) for both cementitious composites, along with their clear tendency to evolve (decreasing contact angle, increasing SFE), suggests that wetting the cementitious surface indirectly induces a noticeable increase in hydrophilicity. In other words, the placement of an additional droplet in the exact location of the previous one would likely result in an enhanced hydrophilic behaviour of the cementitious surface.

### 3.2. Analysis of the Self-Cleaning Properties of Cementitious Composite Surfaces

As shown in the scientific literature, the self-cleaning properties of materials can be determined through a test assessing the decomposition of organic dyes, one of which is Rhodamine B. In general, these reports indicate significant effects in terms of the bleaching capacity of the dye. However, due to the qualitative or semi-quantitative nature of the research methodology, it is difficult to quantify these results, with most studies typically reporting the UV exposure time interval after which bleaching effects become visible.

**Figure 8:**
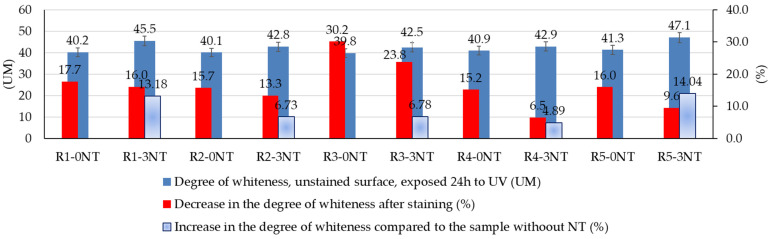
Degree of whiteness of samples before staining—influence of NT on it and its reduction by staining.

**Figure 9:**
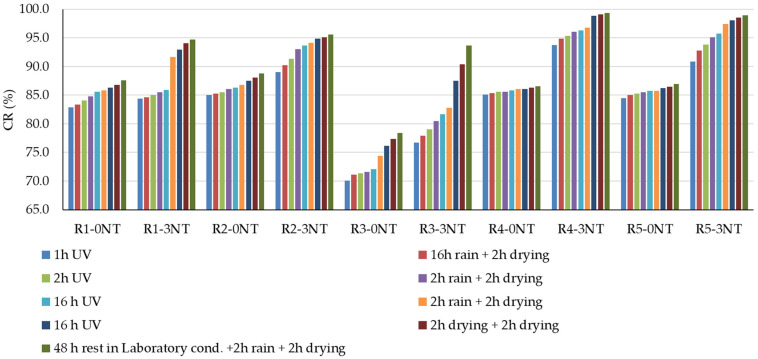
Recovery capacity of the white grade.

For this study, the experimental results showing the evolution of the degree of whiteness of the cementitious composite surfaces stained with Rhodamine B solution are shown in Figure 8, while the capacity for whiteness recovery is shown in Figure 9.

As illustrated in Figure 8, several factors influence the degree of whiteness of the surface of cementitious composites. The partial substitution of natural aggregates with recycled waste aggregates led to a slight variation in the degree of whiteness, ranging from 0.1 to 1.2 units of measure (UM) for composites prepared without the addition of TiO_2_ nanoparticles (R1-0NT to R5-0NT). This parameter remained within the limits of 40.1 for the sample with the lowest degree of whiteness, R2-0NT, and 41.3 for the sample with the highest degree of whiteness, R5-0NT. This minimal variation can be explained by both the majority of the surface consisting of the cementitious binder, which did not cause colour changes as its quality and quantity remained constant, and the nature of the aggregates, which can introduce differences in porosity and roughness that can themselves induce the observed variation [59,60].

However, it can be assumed that the nature of the aggregates is likely to have an indirect effect on the performance of the composites. This conclusion is supported by the observation that aggregates which contribute to a reduction in the water absorption of the composite—whether due to the inherent properties of the aggregate, as is the case with recycled glass aggregates (RGA), or by promoting reduced porosity, as is the case with slag aggregates (GBA) [59,60]—exhibit less whiteness loss after the staining process. Thus, immediately after staining with a constant amount of staining agent, there was a reduction in the degree of whiteness of 15.7% (R2-0NT)/13.3% (R2-3NT) in the case of RGA or 15.2% (R4-0NT)/6.5% (R4-3NT) in the case of GBA, as shown in Figure 8. On the other hand, brick aggregates, which by nature have a higher water absorption, led to a greater reduction in the degree of whiteness of the composite, namely, by 30.2% (R3-0NT)/23.8% (R3-3NT). The reduction in the degree of staining for the R1 composition, which contains only natural aggregates (with varying amounts of TiO_2_ nanoparticles added), fell within intermediate values, namely, 17.7% (R1-0NT)/16% (R1-3NT). This observation suggests that the inherent properties of the materials used to prepare the composites exert a nuanced but discernible influence on the resulting microstructure. This in turn manifests itself in varying degrees of surface staining. The particular case of composites incorporating textolite is noteworthy and warrants further investigation. In spite of the fact that the inclusion of RTA induces remarkable structural changes at the microstructural level and shows an increased water absorption within the composite [59,60], from the point of view of the reduction of whiteness after staining, values closer to those of composites with reduced water absorption were recorded: 16% (R5-0NT)/9.6% (R5-3NT), as shown in Figure 8. This behaviour may indicate that not only the water absorption and porosity of the composite influence its interaction with the staining agent, but also the specific properties of the materials involved. In this case, given that RTA is originally a plastic material, it is highly likely that the staining agent has reduced adhesion to this waste material, resulting in minimised staining due to Rhodamine B retention.

Another factor influencing the degree of whiteness of the surfaces was identified as the NT content in the composite matrix. As can be seen in Figure 8, the inclusion of NT in the composite matrix resulted in an increased degree of whiteness in the composite surfaces compared to the analogous sample prepared without NT for all types of aggregates. This behaviour was anticipated based on references in the literature [52,54], highlighting the use of TiO_2_ as a whitening agent for a wide range of materials, from paints to products in the cosmetics and pharmaceutical industries. Although the amount of NT used as an additive was the same for all compositions (3% by weight relative to the amount of cement, the amount of cement being kept constant), it was observed that the increase in the degree of whiteness was influenced by the type of aggregates derived from waste that replaced NA, ranging from 4.89% for GBA to 14.04% for RTA. It is therefore emphasised once again that both the nature of the aggregates and the presence or absence of NT in the composition are interdependent factors influencing the degree of whiteness of cementitious surfaces. As a result of these reactions, the dye molecule is broken down into progressively smaller molecules, making it more susceptible to removal by the rinsing water. At the same time as this improvement in washing performance by reducing the size of the dye molecules, there is an increase in the hydrophilicity of the surface, as explained in Section 3.1. The water droplets assume a flattened configuration (forming “mini squeegees”), culminating in the ultimate self-cleaning effect. This understanding of the self-cleaning mechanism is further supported by experimental results which indicate that each stage of the test protocol has a distinct effect on the behaviour of the surface.

Looking at the ability of the composites to return to their original state, quantified by the “Recovery Capacity” (CR) indicator, Figure 9 shows both the effect of the amount of NT introduced into the composite matrix and the influence of the aggregates used. First of all, for all compositions, regardless of the type of aggregates used, the presence of NT in the composite matrix correlates with an increased potential for whiteness recovery and restoration to the original unstained state, outperforming compositions without NT. This phenomenon is supported by existing literature [14], which highlights a mechanism involving the degradation of organic molecules through redox reactions initiated by electrons and holes emitted by NT upon activation, facilitated by the energy of UV radiation. Therefore, the colour shift following UV exposure is generally not as pronounced as the discolouration observed following exposure to artificial rain. Considering that artificial rain is always preceded by UV exposure in the test sequence, it is apparent that the self-cleaning process initially involves the photoactivation mechanism of NT, which initiates the degradation of the dye molecule. Simultaneously, the mechanism of increased surface hydrophilicity facilitates the flattening of the water droplets, and subsequently these water droplets act like “squeegees”, increasing the efficiency of dye particle removal compared to their spherical counterparts.

**Figure 10:**
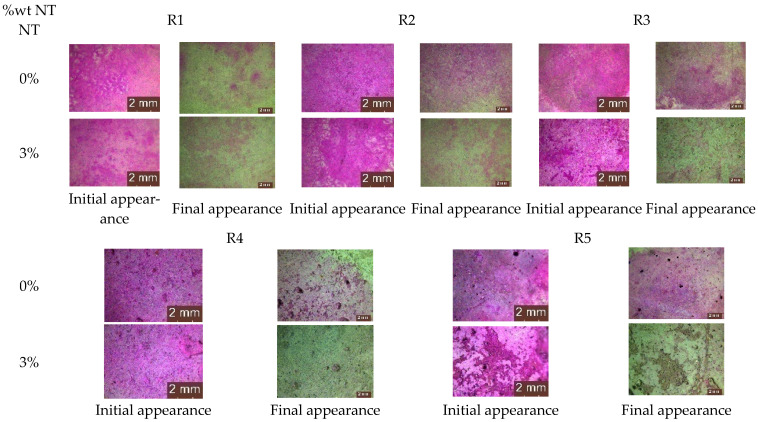
The aspect of the stained area before and after completion of the test cycle—illustrative microscopic images.

In contrast to compositions containing only natural aggregates (R1), those containing ceramic brick aggregates (R3) showed a reduced capacity for dye stain removal. At the end of the testing protocol, as shown in Figure 4, the quantification of the degree of whiteness recovery (CR) showed values of 87.6% for R1-0NT and 94.7% for R1-3NT, as opposed to 78.4% for R3-0NT and 93.6% for R3-3NT. It is therefore clear that NT contributes to the increase in CR, but the substitution of natural aggregates with RBA reduces this effect. On the other hand, composites formulated with recycled aggregates derived from waste glass and NT (R2-3NT), with a CR of 94.9%, or slag and NT (R4-3NT), with a CR of 99.3%, are characterised by superior compactness and reduced water absorption capacity [59,60], which improves the retention of the colouring agent on the composite surface. This property facilitates the subsequent decomposition and removal process. A reduced retention of the staining solution in the composite layer was observed in the case of the composition containing recycled textolite aggregates and NT (R5-3NT). At the end of the test procedure, a CR indicator of 98.9% was recorded. A plausible explanation for this observation lies in the inherent resistance of textolite to the staining agent, as previously discussed. These findings are supported by the microscopic analysis images obtained from the stained areas of the cementitious composite surfaces, as shown in Figure 10.

Therefore, in terms of self-cleaning efficacy, using a Rhodamine B staining method and subjecting the samples to sequential UV exposure-wash cycles, it was observed that the TiO_2_ nanoparticles (NTs) effectively conferred a self-cleaning ability to the composite material, with surfaces partially recovering their original whiteness. This recovery was dependent on both the amount of TiO_2_ nanoparticles present in the composition and the nature of the aggregates. The replacement of natural aggregates with those derived from recycled waste, together with the properties of these recycled aggregates and the incorporation of TiO_2_ nanoparticles, led to a series of microstructural changes within the binder matrix. Furthermore, the depth of penetration of the colouring agent into the cementitious matrix varied from case to case. Consequently, the challenge of its removal under the influence of the wash water also showed variability in different scenarios. Simultaneously, differences in the concentration of TiO_2_ nanoparticles (NT) within the matrix contributed to different degrees of self-cleaning efficiency. With regard to the compositions without NT, no discernible differences were observed due to the partial substitution of natural aggregates (NA) by those derived from recycled waste, suggesting that this parameter is mainly influenced by the properties of the binding matrix. However, it can be argued that recycled aggregates, characterised by increased water absorption, have a modest effect on reducing the self-cleaning potential of the composite surface. Conversely, the uniform distribution of NT throughout the composite matrix is critical. This assertion is supported by the comparison between R1-4NT and R1-5NT, where an increased NT concentration does not induce improvements. Rather, this phenomenon is attributed to the presence of localised NT agglomerates or regions of NT deficiency.

### 3.3. Analysis of the Mould Resistance of Cementitious Composite Surfaces

#### 3.3.1. Cementitious Samples Exposed 720 h to an Environment Contaminated with *Aspergillus niger* Showed the following Developments

**Figure 11:**
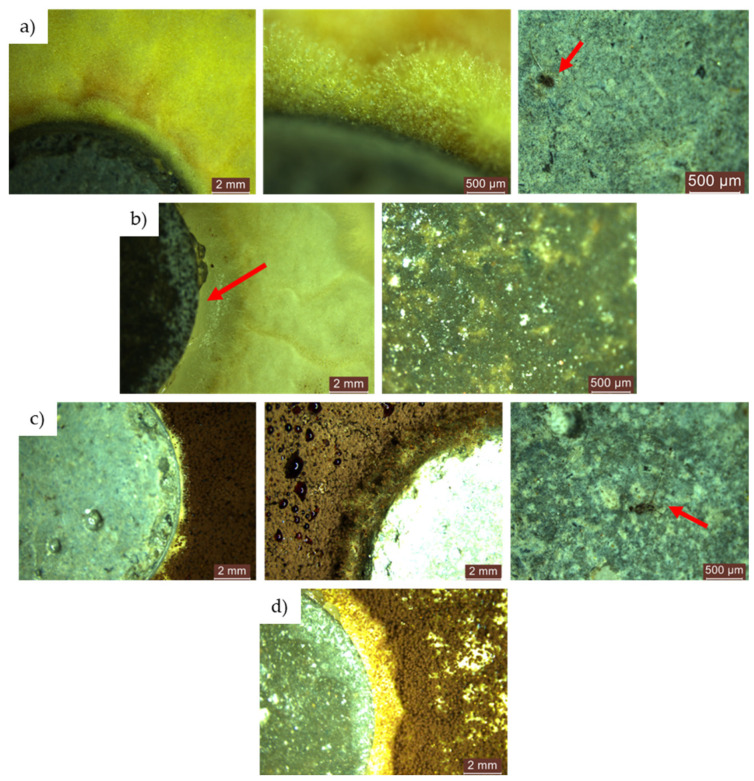
Microscopic analysis of the control cementitious composite sample (R1) exposed in an environment contaminated with concentrated solution (S1) of *Aspergillus niger*: (**a**) composition without NT, exposure 24 h; (**b**) composition with 3% NT, exposure 24 h; (**c**) composition without NT, exposure 48 h; (**d**) composition with 3% NT, exposure 48 h. In subfigures (**a**,**c**) the red arrows indicate the colonies of mould grown on the surface of the samples, while in figure (**b**) the arrow marks the inhibition halo around the sample.

**Figure 12:**
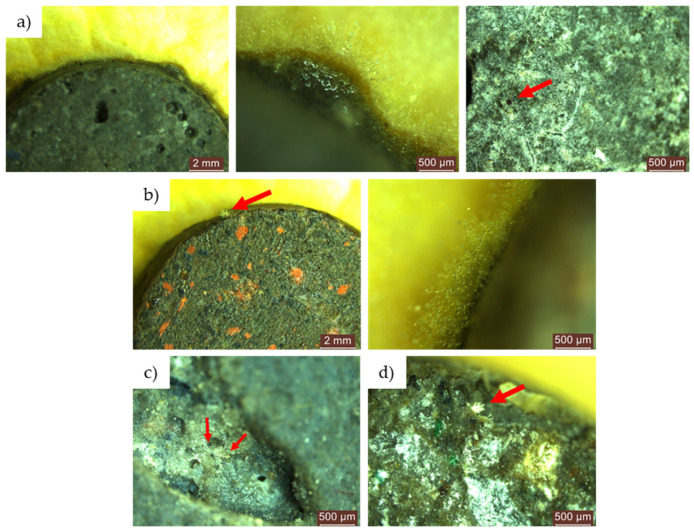
Microscopic analysis of cementitious composite samples containing recycled waste aggregates (R2-0NT… R5-0NT), without addition of NT, exposed for 24 h in a contaminated environment to a concentrated solution (S1) of *Aspergillus niger*: (**a**) composition containing RGA (R2-0NT); (**b**) composition containing RBA (R3-0NT); (**c**) composition containing GBA (R4-0NT); (**d**) composition containing RTA (R5-0NT). The red arrows indicate the colonies of mould grown on the surface of the sample.

**Figure 13:**
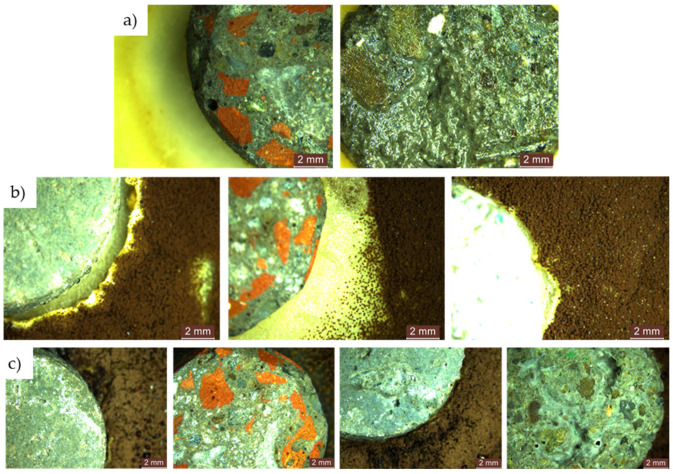
Microscopic analysis of cementitious composite samples with recycled waste aggregates and 3% NT addition (R2-3NT… R5-3NT) exposed to: (**a**) 24 hours in a contaminated environment with concentrated solution (S1) of *Aspergillus niger*—from left to right: composition with RGA (R2-3NT), composition with GBA (R4-3NT); (**b**) 48 hours in a contaminated environment with concentrated solution (S1) of *Aspergillus niger*—from left to right: composition with RGA (R2-3NT), composition with RBA (R3-3NT), composition with RTA (R5-3NT); (**c**) 72-720 hours in a contaminated environment with concentrated solution (S1) of *Aspergillus niger*—from left to right: composition with RGA (R2-3NT), composition with RBA (R3-3NT), composition with GBA (R4-3NT), composition.

In the instances where the concentrated contamination solution (S1) was employed, we administered the following in substantial quantity (via dripping):

- After 24 h of exposure, the control sample (R1-0NT) already showed uniform mould growth along its periphery, accompanied by a clear point of mould growth on its surface (Figure 11a). Conversely, in the case of the mixture containing natural aggregates and a 3% NT additive (R1-3NT), there was no evidence of mould growth along the edges or on the surface of the specimen within the first 24 h of exposure to the contaminated environment. In addition, a subtle inhibition halo with an average size of 1.50–2.50 mm was observed peripherally around the cementitious specimen, coinciding with the absence of mould growth in this region (Figure 11b).

- During the 48 to 72 to 720 h exposure period in the contaminated environment, the control sample (R1-0NT) showed continued mould growth on both the cementitious surface and the edges of the specimen, facilitating spore formation (Figure 11c). Conversely, the R1-3NT sample maintained an inhibition halo that gradually diminished over time, maintaining a clean surface with no traces of mould (Figure 11d).
- Similarly, exposure of the NT-free specimens, derived from cementitious compositions with partial replacement of NA with recycled waste aggregates (R2-0NT… R5-0NT), showed mould proliferation along the edges and evidence of mould presence on the surfaces of the specimens after 24 h exposure to an environment contaminated with *Aspergillus niger*. This trend continued up to 720 h, as illustrated in Figure 12. Notably, an anomaly to this pattern was observed in samples where NA has been partially replaced with GBA (R4), as no evidence of mould presence was detected on either the edges or surfaces of the samples.

- After 24 h exposure, specimens made from cementitious compositions with partial substitution of NA with recycled waste aggregates and the addition of 3% NT (R2-3NT… R5-3NT) showed clean edges and surfaces, free from mould. In addition, inhibition halos were present, the size of which varied between compositions, probably influenced by the nature of the recycled aggregates replacing NA, as shown in Figure 13a.
- Prolonged exposure of these composites (R2-3NT… R5-3NT) containing recycled aggregates and NT to the contaminated environment, as shown in Figure 13b,c, demonstrated the continued resistance of the surfaces to mould activity, with the surfaces remaining clean. However, a reduction in the inhibition halo was observed over time, indicating a decrease in antimicrobial activity within the substrate.

**Figure 14:**
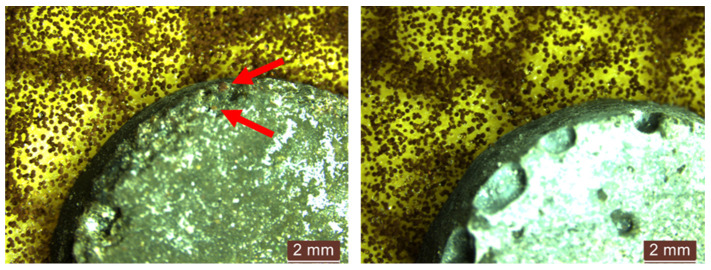
Microscopic analysis of the cementitious composite sample with natural aggregates exposed for 48 h to a contaminated environment with a concentrated solution (S1) of *Aspergillus niger*. (**left**)—composition without NT (R1-0NT), (**right**)—composition with 3% NT (R1-3NT). The red arrows indicate the colonies of mould grown on the surface of the sample.

**Figure 15:**
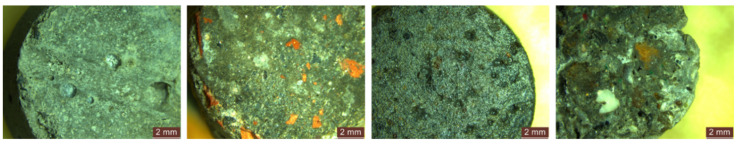
Microscopic analysis of cementitious composite samples with recycled aggregates, containing 3% NT, exposed for 48 to 720 h to a contaminated environment with a concentrated solution (S1) of *Aspergillus niger*—from left to right: composition with RGA (R2-3NT), composition with RBA (R3-3NT), composition with GBA (R4-3NT), composition with RTA (R5-3NT).

When the diluted inoculation solution (S2) was utilised, administered in limited quantity through spraying, the following occurred:

- A proliferation of moulds with a much wider and more uniform spatial distribution was observed on the PDA medium. However, in contrast to the samples contaminated using droplets of the concentrated solution (S1), the population density was much lower.
- Within the first 24 h of exposure, there was no evidence of the presence of mould, except within the open area of the test apparatus, specifically on the surface of the PDA medium.
- After 48 h, the R1-0NT composition (with natural aggregates, no NT) showed initial signs of mould development on both the edges and surface of the specimen, as shown in Figure 14 (left). In contrast, the similar composition with 3% NT (R1-3NT) maintains clean edges and surface without the formation of an inhibition halo, as shown in Figure 14 (right). This condition persists up to the evaluation at 720 h of exposure, with continued mould growth observed in the NT-free sample (R1-0NT).
- Throughout the evaluation period, both in composites with recycled aggregates without NT (R2-0NT… R5-0NT) and those with a 3% NT addition (R2-3NT… R5-3NT), the edges and surfaces of the specimens remained free of mould growth, as shown in Figure 15. However, the presence of a clearly defined inhibition halo was not observed.

Therefore, in addition to the nature of the raw materials (in the cases studied, the nature of the natural aggregates or those derived from recycled waste), another factor that may influence the resistance of cementitious composites in environments contaminated with *Aspergillus niger* mould is the presence and concentration of TiO_2_ nanoparticles in the composite matrix, as well as the concentration of the inoculation solutions, which actually represents the degree of environmental aggressiveness. Specifically with regard to *Aspergillus niger*, the attack on cementitious composites is facilitated by a significant number of infectious propagules present in the environment, a phenomenon accentuated by the use of a concentrated solution (S1) administered in large quantities by dripping.

#### 3.3.2. The Cementitious Samples Exposed for 720 h in an Environment Contaminated with *Penicillium chrysogenum*

**Figure 16:**
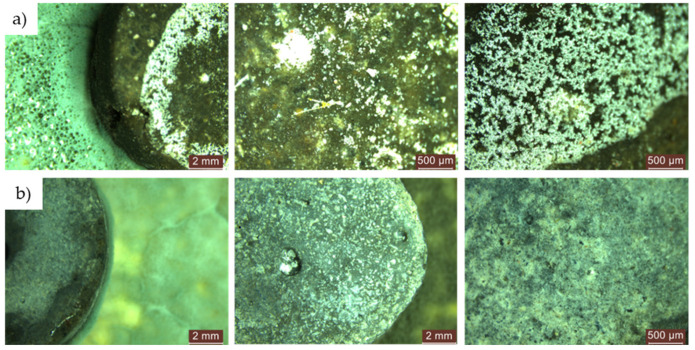
Microscopic analysis of cementitious composite R1 samples exposed for 48 to 720 h to a contaminated environment with a concentrated solution (S1) of *Penicillium chrysogenum*: (**a**) without NT (R1-0NT), (**b**) with 3% NT (R1-3NT).

**Figure 17:**
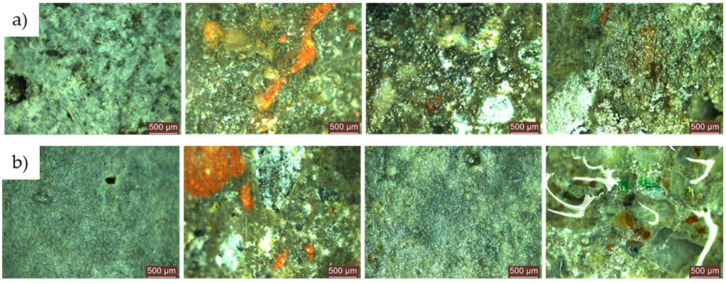
Microscopic analysis of cementitious composite samples with recycled aggregates (R2-R5) exposed for 720 h to a concentrated solution (S1) of *Penicillium chrysogenum* in a contaminated environment—from left to right: composition with RGA (R2), composition with RBA (R3), composition with GBA (R4), composition with RTA (R5). (**a**) Compositions without NT; (**b**) compositions with 3% NT.

In the instances where the concentrated contamination solution (S1) was employed, we administered the following in substantial quantity (via dripping):

- The cementitious composites containing natural aggregates (R1) showed an inability to induce the formation of an inhibition halo, despite the inclusion of NT in the composition. In cases where NT was absent from the composition (R1-0NT), mould colonies proliferated rapidly on both the surface and edges of the specimens within the first 48 h of exposure to the contaminated environment, as shown in Figure 16. These colonies progressively increased in size with prolonged exposure, as observed in Figure 16a. Conversely, there was no discernible evidence of mould development on the cementitious surface in compositions containing NT throughout the 720 h evaluation period, as shown in Figure 16b.

- Within the initial 48 h exposure period in a contaminated environment, cementitious composites containing recycled aggregates showed no evidence of mould growth, irrespective of the presence or absence of NT in the composite matrix. This remained consistent throughout the 720 h observation period, as shown in Figure 17.

**Figure 18:**
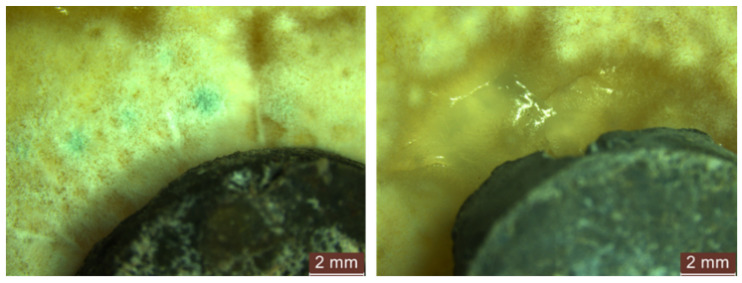
Microscopic analysis of cementitious composite specimens R1 exposed for 48 h to a contaminated environment with a diluted solution (S2) of *Penicillium chrysogenum*—without NT, R1-0NT (**left**); with 3% NT, R1-3NT (**right**).

**Figure 19:**
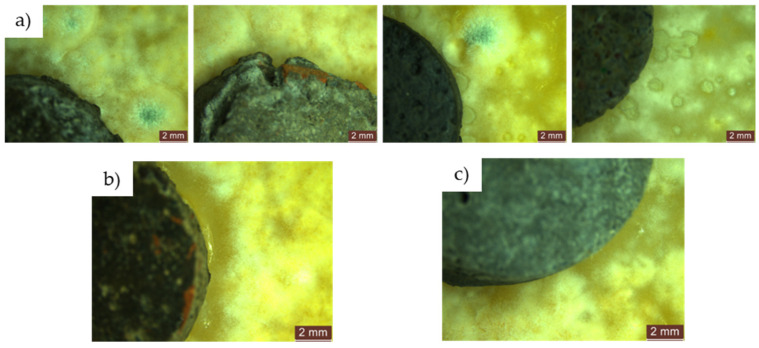
Microscopic analysis of the cementitious composite samples R2-R5 exposed for 48 h to a medium contaminated with a dilute solution (S2) of *Penicillium chrysogenum*. (**a**) Composites without NT—from left to right: composition with RGA (R2), composition with RBA (R3), composition with GBA (R4), composition with RTA (R5). (**b**) Inhibition halo developed around the R3 composite sample with 3% NT. (**c**) Inhibition zones developed around the R2 composite sample with 3% NT.

When the diluted inoculation solution (S2) was utilised, we administered the following in limited quantity through spraying:

- The cementitious composite containing natural aggregates without NT (R1-0NT) did not show the formation of an inhibition halo, as illustrated in Figure 18 (left). In contrast, the inclusion of 3% NT led to the observation of an inhibition halo with an average size of 1–1.5 mm after 48 h exposure to the contaminated environment, as shown in Figure 18 (right). There was no evidence of mould growth on the surface of the samples, and these states held throughout the 720 h period.

- For cementitious composites with recycled aggregates and without NT (R2-0NT… R5-0NT), no inhibition halo was observed around the specimens, as shown in Figure 19a. However, for composites containing NT (R2-3NT… R5-3NT), an inhibition halo was present, especially when natural aggregates were replaced by brick waste aggregates (R3-3NT), with an average size of 0.8–1.5 mm, as shown in Figure 19b.

- When natural aggregates were replaced with glass waste (R2-3NT), slag (R4-3NT), or textolite (R5-3NT), no continuous inhibition halo was observed around the specimens. Instead, irregular zones of inhibition were observed adjacent to the samples. This condition persisted up to the evaluation carried out after 720 h of exposure to an aggressive environment, with no sign of mould growth on the specimen surfaces. Thus, for this type of mould, the tested materials exhibited an irregular inhibitory effect in the environment that could persist for long periods.

Thus, similar to the exposure to *Aspergillus niger*, it can be concluded that the resistance of cementitious composites to the attack of *Penicillium chrysogenum* is influenced by several factors: the nature of the aggregates (in the cases studied, whether natural aggregates or those derived from recycled waste), the presence of TiO_2_ nanoparticles in the composite matrix, and the concentration of the inoculants.

#### 3.3.3. Microscopic Analysis of the Specimen Surfaces after 120 Days (4 Months) of Exposure to the Contaminated Environment

**Figure 20:**
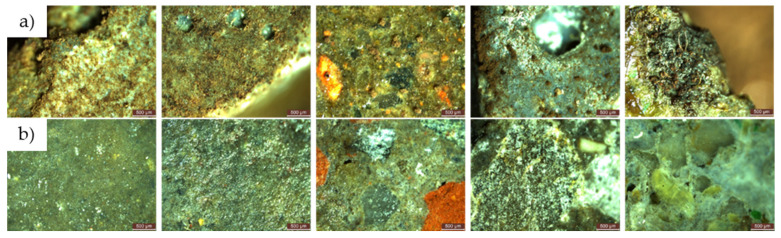
Microscopic analysis of cementitious composite samples after 4 months of exposure to a concentrated solution (S1) of *Aspergillus niger* to a contaminated environment—from left to right, the control composition with natural aggregates (R1), the composition with recycled glass aggregate (R2), the composition with recycled brick aggregate (R3), the composition with glass aggregate (R4), the composition with textolite (R5): (**a**) compositions without NT; (**b**) compositions with NT.

**Figure 21:**
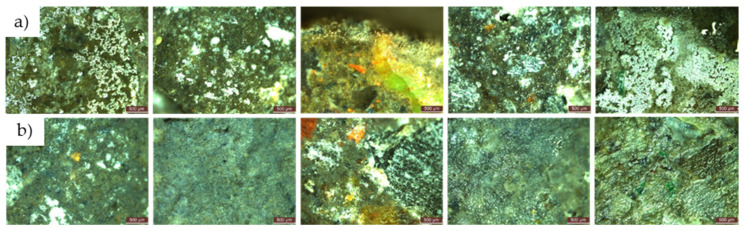
Microscopic analysis of cementitious composite samples after 4 months of exposure to a concentrated solution (S1) of *Penicillium chrysogenum* to a contaminated environment—from left to right, the control composition with natural aggregates (R1), the composition with recycled glass aggregate (R2), the composition with recycled brick aggregate (R3), the composition with glass aggregate (R4), the composition with textolite (R5): (**a**) compositions without NT; (**b**) compositions with NT.

**Figure 22:**
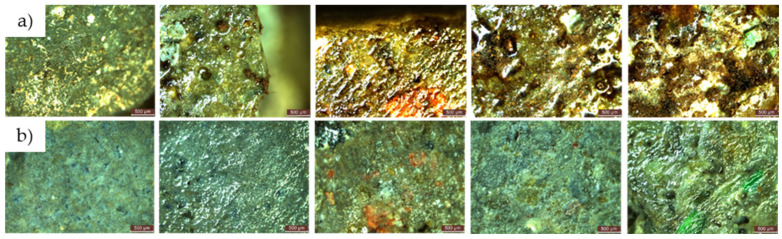
Microscopic analysis of cementitious composite samples after 4 months of exposure to a diluted solution (S2) of *Aspergillus niger* to a contaminated environment—from left to right, the control composition with natural aggregates (R1), the composition with recycled glass aggregate (R2), the composition with recycled brick aggregate (R3), the composition with glass aggregate (R4), the composition with textolite (R5): (**a**) compositions without NT; (**b**) compositions with NT.

**Figure 23:**
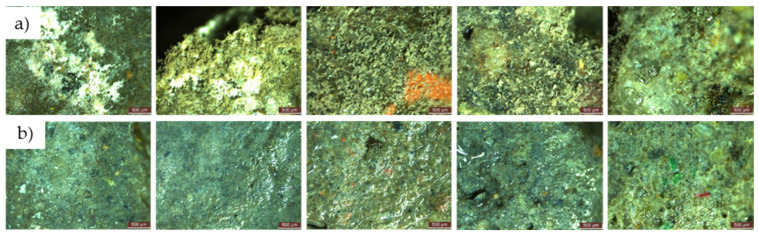
Microscopic analysis of cementitious composite samples after 4 months of exposure to a diluted solution (S2) of *Penicillium chrysogenum* to a contaminated environment—from left to right, the control composition with natural aggregates (R1), the composition with recycled glass aggregate (R2), the composition with recycled brick aggregate (R3), the composition with glass aggregate (R4), the composition with textolite (R5): (**a**) compositions without NT; (**b**) compositions with NT.

- For both types of mould, *Aspergillus niger* and *Penicillium chrysogenum*, evidence of mould colony development was observed on the surface of the R1–R5 cementitious composite specimens that did not contain NT, regardless of whether the concentrated inoculation solution (S1) (Figure 20 and Figure 21) or the diluted solution (S2) was used (Figure 22 and Figure 23).

When comparing each composition, using a semi-quantitative assessment of mould proliferation indicators (for both mould species used), it was apparent that when using a more dilute inoculation solution (S2 variant), coupled with the application of a reduced solution volume resulting in a 60-fold reduction in inoculum concentration, there was a significant reduction in mould colony density. It can therefore be hypothesised that these cementitious composites are susceptible to surface colonisation by *Aspergillus niger* or *Penicillium chrysogenum*, albeit influenced by the concentration of the available inoculum. Moreover, within the framework of these non-NT cementitious composites, a horizontal comparative analysis showed that certain aggregates derived from recycled waste facilitate the proliferation of moulds (most notably observed in the case of textolite—R5), while others inhibit such proliferation (most remarkably observed in the case of brick and slag—R3 and R4), in contrast to the reference sample R1 (without recycled aggregates), keeping constant other parameters such as the type of mould, the type and quantity of inoculation solution, and the duration of exposure.

- No evidence of colonisation was observed on the surfaces of cementitious composite samples R1–R5 containing 3% NT (3% by weight relative to the amount of cement) when using either concentrated (S1) or dilute (S2) inoculants. However, it is noteworthy that even in cases where an inhibition halo or inhibition zones were initially observed around the specimens, these phenomena largely dissipated over time, with the mould film extending to the interface region between the cementitious specimen and the PDA nutrient substrate.

Therefore, the resistance of cementitious composites to mould attack is confirmed both in the case of exposure to *Aspergillus niger* and in the case of exposure to *Penicillium chrysogenum*, an observation that is consistent with existing reports in the literature [14,61,62]. Nevertheless, it can be stated that this resistance is influenced by the inherent characteristics of the raw materials (specifically, the nature of the natural aggregates or those derived from recycled waste) in the cases studied, together with the presence and concentration of TiO_2_ nanoparticles in the composite matrix and the concentration levels of the inoculation solutions. The incorporation of TiO_2_ nanoparticles into the cementitious matrix of the composites significantly increases their resistance to mould attack, leading to the acquisition of biocidal capacity by the cementitious surface. However, these cementitious composites, despite their improved performance due to the addition of NT, do not exhibit a biocidal effect beyond the perimeter of the specimens. This observation, when compared with previous investigations documented in the scientific literature, has led to the generation of new research hypotheses. Notable differences from previous studies include the use of white Portland cement in the previous experimental framework and the exclusive inclusion of NT in the cement paste samples.

In this context, the cement employed was grey Portland cement (white Portland cement in production contains a higher TiO_2_ content), while the specimens were fashioned from a cementitious composite encompassing both cement and aggregates. Thus, despite maintaining the NT content at 2–5% (by mass relative to the cement quantity), the inclusion of both cement and aggregates in the composition resulted in an overall lower NT concentration, leading to a diminished biocidal potency. Essentially, this inadequacy falls short in inhibiting mould growth in the marginal zones of the specimen peripheries, thereby failing to establish an inhibition halo.

Microscopic analysis shows that the incorporation of TiO_2_ nanoparticles (NT) into the composition imparts a biocidal capacity to the surface of the material. This capacity is influenced by the NT concentration and the type of aggregates used. Under constant conditions, including mould type, inoculant type and amount, and exposure time, certain recycled waste aggregates were found to promote mould growth, particularly in the case of textolite (R5). Conversely, other aggregates, such as those derived from brick and slag (R3 and R4), inhibit mould growth compared to the control sample R1.

Furthermore, in the case of exposure of samples to mould-contaminated environments, for both *Aspergillus niger* and *Penicillium chrysogenum*, two other influencing factors have been identified: the duration of exposure and the aggressiveness of the contaminating environment (represented by the concentration and amount of inoculation solution used). In general, the development of mould colonies is favoured by longer exposure and higher concentrations of the inoculation solution.

Another influential factor in terms of self-sanitising capacity is the type of mould. The data analysed suggest that *Penicillium chrysogenum* has a slower colonisation rate on composite samples in general (requiring a longer exposure time in the contaminated environment for the first signs of growth to appear on the edge or surface of the sample) and is particularly slow to colonise composite samples without NT. In contrast, *Aspergillus niger* shows easier colonisation on the material, with initial signs appearing after 24 h of exposure. Once established, it shows greater resistance, but its surface spread is more difficult and remains localised compared to *Penicillium chrysogenum*, which tends to spread more vigorously once it has colonised the area.

For the improved analysis of mould presence and dynamics in relation to cementitious samples, we propose a new concept entitled *Indoor Environmental Agressivity (IEA)*. This concept allows a fast analysis of the samples in relation to indoor microflora and can be defined as the mould population and dynamics in relation to tested samples. It is an approach that enables the use of different inoculation techniques to simulate IEA, maintaining the levels for results interpretation.

The resistance of cementitious composites is observed on a three-level scale which explains three possible situations:

1. compete biocide effect—the sample does not present any fungal contamination; a halo is clear and visible around the cementitious samples;
2. partial biocide effect—the sample present mould marks or weak contamination; the halo is partial visible around the cementitious samples, and they have an irregular shape;
3. no biocide effect—the samples are completely covered by moulds; no halo (partial or complete) is visible around the cementitious samples.

## 4. Conclusions

The objective of this experimental investigation was to evaluate the self-cleaning and self-sanitising performance of cementitious composites in which natural aggregates were partially replaced with recycled waste aggregates supplemented with titanium dioxide nanoparticles. The results of the experimental investigation showed that both the composition of the aggregates (whether natural or partially replaced with recycled waste aggregates) and the inclusion of TiO_2_ nanoparticles in the composite structure are key determinants affecting both the self-cleaning ability of the surface and its resistance to mould infestation such as *Aspergillus niger* or *Penicillium chrysogenum*. Consequently, the following conclusions can be drawn:

The experimental results indicate that the surfaces of cementitious composites exhibit hydrophilic properties as a consequence of the incorporation of NT and their subsequent photoactivation. The study also shows that TiO_2_ nanoparticles confer a self-cleaning ability to composite materials, with surfaces partially recovering their original whiteness, a process influenced by the concentration of NTs and the nature of the aggregates. In addition, the uniform distribution of NTs is critical for effective self-cleaning, as higher NT concentrations do not always lead to improvements due to possible localised agglomerates or NT-deficient regions. Additionally, the NT concentration and the nature of the aggregates influence the biocidal capacity imparted to the surface through the introduction of TiO_2_ nanoparticles. Under controlled conditions, some recycled waste aggregates, such as textolite (R5), promote mould growth, while others, such as brick and slag (R3 and R4), inhibit it compared to the control sample R1.

Furthermore, mould colony growth on composite samples is influenced by exposure time and inoculum concentration, with longer exposure and higher concentrations favouring mould development. In addition, *Penicillium chrysogenum* was observed to colonise more slowly, particularly on samples without TiO_2_ nanoparticles, while *Aspergillus niger* was observed to colonise more rapidly but tended to remain localised once established, in contrast to the more widespread growth of *Penicillium chrysogenum*.

Therefore, in light of the results presented, these investigations have made a significant scientific contribution, supporting the argument for the incorporation of NT in cementitious composites to provide surfaces with enhanced self-cleaning and self-sanitising properties. Such findings are of significant importance, particularly in terms of enhancing the longevity of structures, especially their surface finishes, by reducing the risk of degradation due to biofilm formation. At the same time, they facilitate the maintenance of a high level of safety and hygiene in the environment, with a positive impact on public health. Furthermore, notwithstanding the potential influence of raw material properties on the self-cleaning and self-sanitising capabilities of cementitious composites, particularly the nature of the aggregates in these cases, it can be argued that the inclusion of NT in the composition indirectly improves the feasibility of using recycled waste aggregates while maintaining hygiene standards. In addition, providing surfaces with self-cleaning properties helps to reduce the maintenance requirements of the structures.

## Figures and Tables

**Table 4:** Control recipe (R1).

Recipe Code	Design Class	Water/ Cement Ratio	Cement (kg/m^3^)	Natural Aggregates, Cumulative (kg/m^3^)	Natural Aggregates, Sort 0/4 mm (% of Total Aggregates)	Natural Aggregates, Sort 4/8 mm (% of Total Aggregates)	MasterEase 5009 Superplasticiser Additive (% Mass Ratio to Cement Quantity)
R1	C 20/25	0.6	366	1577	70	30	0.5

**Table 5:** Average values of contact angles and STF at the liquid–sample interface.

Sample Code	Average Contact Angle at the t_0_ Moment (°)	Average Contact Angle at the t_100_ Moment (°)	STF at the t_0_ Moment (mN/m)	STF at the t_100_ Moment (mN/m)
R1-0NT	60.87	47.83	47.67	54.99
R1-3NT	22.77	18.15	67.74	69.39

## Data Availability

Data are contained within the article.
